# Selecting and training candidates to suit their role

**Published:** 2018-07-31

**Authors:** Dhivya Ramsamy, Daksha Patel

**Affiliations:** 1 Senior Faculty: LAICO – Aravind Eye Care System, Gandhi Nagar, Madurai, India.; 2 E-learning Director: International Centre for Eye Health, London School of Hygiene and Tropical Medicine, London, UK.

**Training the eye team can improve individual and community eye health – provided the right people are trained, and in the right way.**

**Figure:**
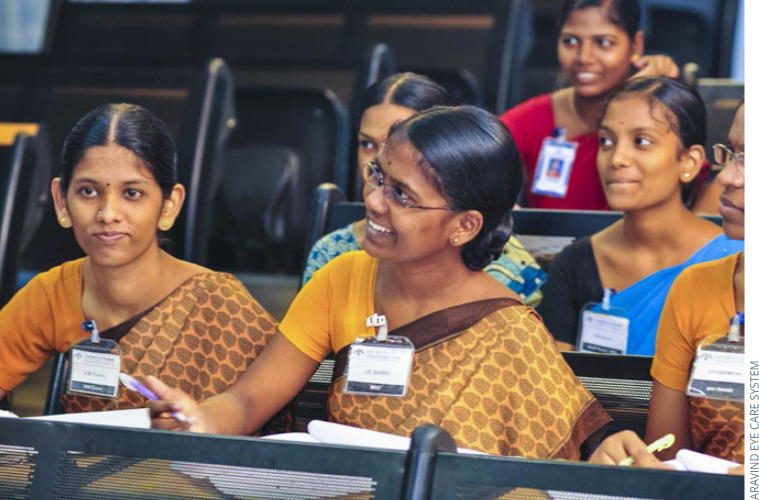
Select and train the best candidates. INDIA

In health care, the quality and training of eye care personnel determine the health outcomes for individual patients and the community.

We can improve the quality of human resources for eye health by understanding the different roles within the eye team, selecting the right people to train for each role, training them well, and continuing their training as needed.

## Understand the different roles

In the eye care team, each role is defined by a job description, which lists the set of **competencies** (tasks and processes) which the person must be able to perform with a specified degree of expertise. For example, a vision technician in India must be competent to perform torchlight examination, retinoscopy, subjective refraction, fundus photography, tonometry, and other specified skills.

**Figure:**
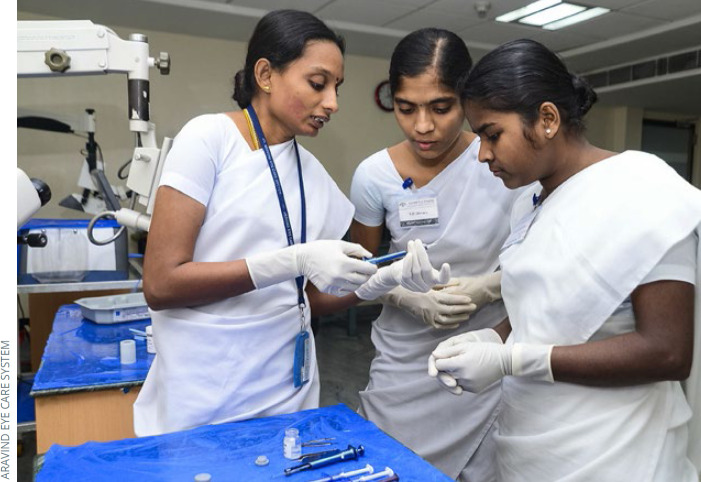
Providing supportive supervision improves learning. INDIA

Investment in training is needed if the required competencies (knowledge and skills) are not available.

## Selection

When selecting candidates to train, consider what will be expected of them in their role once they qualify. What criteria should they be able to meet (e.g. language, past learning achievements, location, gender)? Would their attitude (e.g. compassion, patience and the ability to work as part of a team) suit the role? This is sometimes referred to as their **value fit**.

The criteria for selection must be clearly explained before inviting candidates to apply. Selection must be non-biased and designed to find the best candidate. It can be done based on candidates' curriculum vitae, simple written or practical exercises to identify existing knowledge and skills (e.g. testing visual acuity), and observation of the candidate to assess their attitude.

## Training

Training must be based on the required job description. For each competency, there must be an equivalent ‘learning outcome’ or ‘learning objective’ (these terms are often used interchangeably). For example, the competency ‘Competent to perform torchlight examination’ becomes: ‘By the end of this course, graduates will be able to competently perform a torchlight examination’.

Training programmes must ensure that candidates receive the following:

- All the theoretical knowledge they need to perform the procedure, including safety procedures and contraindications
- Practical supervised instruction and opportunities to practice the procedure
- Suitable opportunities to demonstrate each procedure in a patient-centred manner.

### Training tips

**Figure 1:**
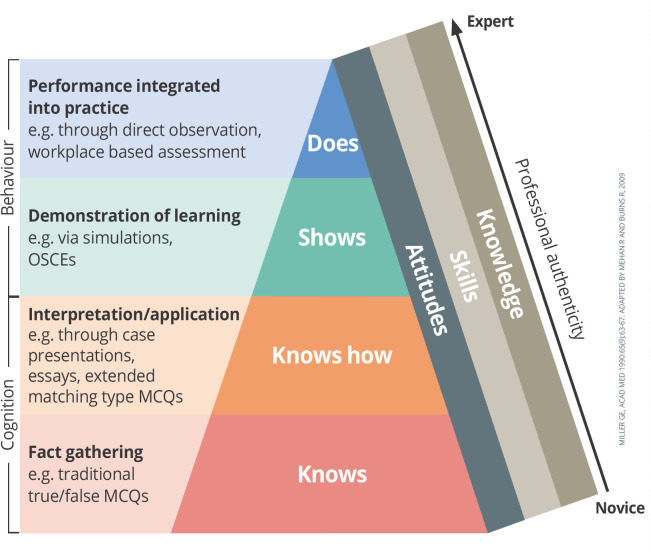
Miller's pyramid: assessing competency and performance

- Draw on candidates' existing capabilities and knowledge
- Give them opportunities to practice and learn from mistakes in a safe learning environment (e.g. by practicing in a wet lab)
- Assess candidates' progress at regular intervals by checking their knowledge and skills against the standards expected of them (Figure 1). Give relevant and accurate feedback that will help them improve (**formative assessment**)
- Practical assessments and examinations at the end of the course or module is designed to objectively determine if the candidate has mastered the taught competencies (**summative assessment**).

### Teaching methods for active learning

How students learn is as important as what they learn. Training should include exposure to real-world or clinical situations. This can be done through observation, using practice labs (e.g. surgical wet labs), or by performing the procedure under supervision. Hospitals that perform their own training should strive to integrate training within patient care.

Today's technology allows for flexible training options such as online courses, videos and webinars to supplement or even replace the classroom.

In the **flipped classroom** model of teaching, trainees are required to engage with the content online before coming to class, and the classroom sessions are typically used to clarify and test their understanding.

In **mixed-mode** or **blended** learning, trainees complete part of the course online, over a set period (while away from the training centre) and then come to the centre to complete their training. This makes better use of trainers' time and learners actively take charge of their learning.

**Figure:**
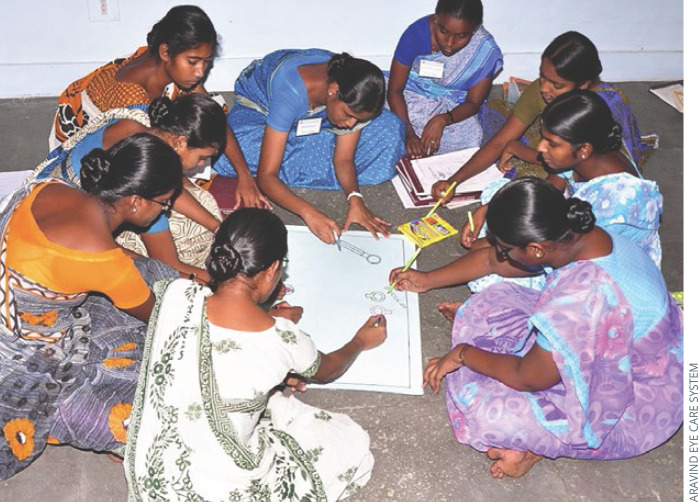
Interactive learning supports the team approach. INDIA

## Ongoing training

Quality is a moving target, and procedures and equipment change as time goes on. Constant retraining is essential to maintain quality. Here are some examples of what to look for when re-assessing training needs.

- Patient safety incidents
- Patient complaints
- New technology
- Changes made to standard protocols and procedures
- Staff performance records (e.g., a surgical outcomes audit may highlight needs for retraining)

We can sustain and improve the quality of human resources for eye care by recruiting the right people and training them to be competent.

**Useful resources for training**

**A framework for allied ophthalmic training programmes (IJCAHPO)**

Contains step-by-step information on the development of training programmes.

**http://documents.jcahpo.org/documents/A_Framework_for_Allied_Ophthalmic_Training_Programs.pdf**

**International core curriculum for refractive error**

**www.icoph.org/resources/268/International-Core-Curriculum-for-Refractive-Error.html**

**International core curriculum for ophthalmic assistants**

**www.icoph.org/resources/31/International-Core-Curriculum-For-Ophthalmic-Assistants.html**

**Useful apps for classroom teaching**

Padlet

Kahoot

Socrative
